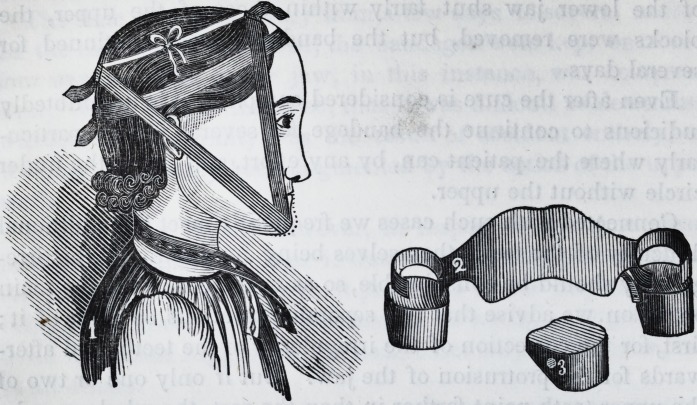# Operation for Correcting Protrusion of the under Jaw

**Published:** 1844-12

**Authors:** 


### ARTICLE XII.

*Operation for Correcting Protrusion of the Under Jaw.* By the SYRACUSE EDITOR.

WE have been led to believe that the attention of the dental profession to this deformity, has not hitherto been commensurate with its importance. There are few irregularities of feature, which produce a greater deformity, and few, we may add, are more under the control of art, when taken seasonably. It is not our present intention to speculate upon the cause of this deformity, but simply to detail such method of treatment as in our hands, has been perfectly successful in restoring the jaw to its natural position. As this position of the jaw is seldom noticed previous to the shedding of the temporary teeth, its occurrence is hence generally considered as being connected with second dentition. This, in a great majority of instances, is doubtless the origin of the difficulty, yet cases are recorded as existing previous to this period. Though such an one has never come under our observation, yet judging from the comparative length of the under jaw, in some cases, which we have observed in adults, we can readily believe it possible.

We have seen a single instance which, from the great length of the under jaw, we not only presume was congenital, but that a remedy could never be effected by art. In this instance the lower jaw could not have been dragged from its natural position by the upper teeth, as the incisors of the lower jaw were at least half an inch anterior to the corresponding ones of upper jaw.

The following case will illustrate the method we have pursued in correcting this deformity. The subject was a daughter of the Rev. Dr. A., of this village, 12 years of age. We first saw her about one year since, and occasionally after, to the time the operation was commenced. During this time the deformity gradually increased—the molars of the two jaws not meeting each other, the upper incisors were constantly acting upon those of the under jaw, throwing it forward. The dotted line, anterior to the chin, in the accompanying cut, (fig. 1,) shows very nearly its projection, at the time of the commencement of the operation. In the treatment of this case we followed, with a few exceptions, the directions of Dr. Gunnell, of Washington City, in his excellent essay upon this subject, read before the American Society of Dental Surgeons, and published in the American Journal of Dental Science, vol. 3, page 65. In this case, we first prepared a *plate*, with *clasps*, in all respects, as we would do with the view of inserting plate teeth.

The clasps were attached to the first permanent molars, (the second not having made their appearance,) and so that the whole could be easily removed and replaced by the patient. To this plate, just within the circle of the clasp, and inside the teeth, were soldered standards, resembling the linings of plate teeth, and passing sufficiently below them to admit of the blocks being attached, which was done by a gold rivet. These blocks were of such thickness as to separate the points of the incisors about one-eighth of an inch. We greatly prefer this method of securing the ivory blocks in their proper position, to that described by Dr. Gunnell, for several reasons. In this way we obviate the difficulty which frequently, and indeed very generally exists, of *securing* the blocks, thus avoiding all risk of their getting misplaced, or any accident which might occur by their getting off, especially during sleep.

The only teeth to which we can apply ligatures, are frequently so short, and of such shape, that it is impossible to secure blocks by ligatures alone, without carrying them so far under the gum, as to do great mischief. We think that ligatures of any kind, and for any purpose as connected with teeth, should always be avoided when practicable. Another important object is also gained by this arrangement; that of enabling the patient to remove them at will, for the purpose of cleansing the mouth and teeth; and the operator, to make any necessary alteration.

It is true that it requires more time to adjust the apparatus in this way, at first, than simply to tie on blocks, yet judging from our own experience, this loss is more than compensated for, by the great ease in removing, and replacing them, as often as is required, to say nothing of its being far more safe, cleanly and less injurious to the teeth which are to support them. This apparatus is represented in fig. 2. The fixture being thus prepared, and adjusted to its place, the bandages are next applied. These are represented in fig. 1, as also the contour of the face after the operation was completed.

As the whole pressure from both bandages comes upon the chin, great care should be taken in adapting a pad for it, which shall be both easy, and *firm*, before the bandages are applied. Pads should also be applied to all points where the bandages produce any irritation.

In relation to the comparative tension of the two straps, it may be observed, that it being the office of the perpendicular one, to overcome the resistance of the parts holding the condyle of the jaw in its bed, and as no progress backward can be made till this is effected, we are of opinion that the tension of the perpendicular strap at first should be fully equal to, if not greater than that made upon the one which extends obliquely backwards.

After this object is, to some extent, attained, the tension of the oblique strap may be gradually increased. It will be perceived, moreover, that the blocks should be placed as far back as possible, for we thus augment the lever power by increasing the length of the long arm, or the space between the fulcrum, or block, and the point at which the power is applied, viz. the end of the chin, and at the same time equally diminish the short arm, or the space between the block and joint of the jaw. The bandages should be removed each day, and the mouth thoroughly cleansed, and the face, particularly about the joint of the jaw, rubbed with some mild liniment. The camphorated liniment, made by dissolving camphor in sweet oil, is perhaps as good as any that can be recommended.

In respect to the time required to perfect a cure, it is greatly varied by circumstances, such as the comparative length of the jaws, age of the patient, &c.

When this deformity is the result of accident, or where the under jaw is of ordinary length, and has been drawn from its natural place by the action of the upper teeth, the condyle of the jaw is, of course, anterior to its natural bed, and requires comparatively but little effort or time to replace it; but where the under jaw is naturally too long to bring its incisors behind those of the upper jaw, a *new socket* is to be formed, which is a much more tedious and protracted operation.

The time may hence vary from a few days to several weeks. In the case above described, the bandages were kept on about four weeks. The under jaw, in this instance, was comparatively very long; so much so, that it was difficult to determine whether the deformity was the result of accident entirely, or whether it had only been augmented by the action of the upper teeth.

It should be stated, however, in respect to the time spent in regulating this case, that it was unnecessarily protracted by an oversight, which, though it produced much perplexity and delay, was, nevertheless, fortunate in its result, as it gave rise to an important suggestion, applicable not only to this case, but to all others.

For the first five or six days after the apparatus was applied, the advance was regular and as rapid as could be expected; but about this time it seemed to resist every effort we could make to move it farther, and it remained in this way for more than a week before the real obstacle to progress was detected. We found, at length, on a close inspection of the mouth, what we had neglected to observe before, that the points of the under teeth, where they rested upon the blocks, very sharp, and as the ivory had gradually softened, these points had become imbedded into it, so as to make the greatest pressure unavailing.

We now altered the blocks in two very important particulars. They were at first of equal thickness from end to end. This shape was changed by filing them nearly to an edge, from front backwards, making them wedge form, with the base looking forward. (See fig. 3.) This constituted an inclined plane, so that the tendency of the pressure, made directly upwards, was to throw the under jaw back. To guard more perfectly still against the contingency, which had been a great source of perplexity, we covered the surface of the blocks, against which the under teeth rested, with gold plate. With this arrangement the progress was astonishingly rapid, and every obstacle to perfect success was removed.

We should advise blocks to be constructed in this way at first. In the above case we have no doubt that it would have been a saving of at least two weeks. As soon as the incisors of the lower jaw shut fairly within those of the upper, the blocks were removed, but the bandages were continued for several days.

Even after the cure is considered complete, it is undoubtedly judicious to continue the bandage for several *nights*, particularly where the patient can, by any effort, still bring the under circle without the upper.

Connected with such cases we frequently meet the additional difficulty of the teeth themselves being irregular. If this irregularity should be considerable, so as to interfere with the main operation, we advise that two separate operations be made of it; first, for the correction of the irregularity of the teeth, and afterwards for the protrusion of the jaw. But if only one or two of the upper teeth point farther in than the rest, the whole may be attempted at the same time. This may be easily accomplished without retarding the main operation, by soldering straps on to the plate at nearly right angles with it, passing down behind the teeth we wish to bring forward, like the lining or back of a plate tooth. This piece must come so far below the point of the tooth as to be caught by the under teeth. The lower extremity would at first require bending forward, so as to shut outside the lower incisors, and so that they will exert due pressure upon it when the mouth is as nearly closed as it may be while the blocks are in their place. By straightening this standard slightly each day, as the tooth yields, we, as a general rule, correct any irregularity of the *teeth*, as soon as the general object is accomplished, or the *jaw* is brought into its proper position.

If the lower point of this gold strap or standard should make undue pressure upon the under tooth or teeth on which it rests, the lower extremity may be widened to any extent, even so as to include *every* under tooth.

It will be readily seen that this, instead of retarding the restoration of the jaw, would tend to facilitate that operation. Indeed, this would be a very convenient method of conducting the main operation, were it not for changing the position of the teeth where they were already symmetrical.

## Figures and Tables

**Figure f1:**